# Population genetics and plant growth experiments as prerequisite for conservation measures of the rare European aquatic plant *Luronium natans* (Alismataceae)

**DOI:** 10.3389/fpls.2022.1069842

**Published:** 2023-01-13

**Authors:** Weronika A. Makuch, Stefan Wanke, Barbara Ditsch, Frank Richter, Veit Herklotz, Julian Ahlborn, Christiane M. Ritz

**Affiliations:** ^1^ Institute of Biology, Geobotany and Botanical Garden, Martin-Luther University Halle, Halle, Germany; ^2^ German Centre for Integrative Biodiversity Research (iDiv) Halle-Jena-Leipzig, Leipzig, Germany; ^3^ Institut für Botanik, Fakultät Biologie, Technische Universität Dresden, Dresden, Germany; ^4^ Departamento de Botanica, Instituto de Biología, Universidad Nacional Autonoma de Mexico, Distrito Federal, Mexico; ^5^ Botanischer Garten der Technischen Universität Dresden, Dresden, Germany; ^6^ Sächsisches Landesamt für Umwelt, Landwirtschaft und Geologie, Dresden, Germany; ^7^ Senckenberg Museum for Natural History Görlitz, Senckenberg – Member of the Leibniz Association, Görlitz, Germany; ^8^ Professur für Biodiversität der Pflanzen, Internationales Hochschulinstitut (IHI) Zittau, Technische Universität Dresden, Zittau, Germany

**Keywords:** aquatic plant, Alismatales, *Luronium natans*, endangered species, population genetics, conservation, growth form

## Abstract

Information provided by population genetic studies is often necessary to effectively protect endangered species. In general, such data is scarce for aquatic plants and this holds also for *Luronium natans*, an aquatic macrophyte endemic to northwestern and western Europe. It is threatened across its whole distribution range due to human influences, in particular due to eutrophication and intensive fish farming. In spite of habitat protection populations continue to decline and re-introductions are one possibility to prevent the species’ extinction. Therefore, insights in genetic diversity and relatedness of source populations is warranted.

Thus, we performed Amplified Fragment-Length Polymorphism (AFLP) on two large populations in Saxony, Germany (*Großenhainer Pflege* and *Niederspree*), complemented with numerous additional occurrences from Europe. In addition, we conducted experiments on plant growth to assess optimal conditions for *ex-situ* cultivation taking water temperature, water level and substrate into account.

We revealed considerably high levels of genetic diversity within populations (Shannon Indices ranged from 0.367 to 0.416) implying that populations are not restricted to clonal growth only but reproduce also by open-pollinated flowers. Remarkably, the two geographically close Saxon populations were genetically distant to each other but subpopulations within a locality were completely intermingled. Concerning optimal cultivation conditions, longest roots were obtained at temperatures >14°C and saturated, but not submerging water levels.

Thus, our findings advocate for a re-introduction scheme from nearby source populations and provide detailed information on successful *ex-situ* cultivation.

## 1 Introduction

The loss of aquatic vegetation has been accelerating in the last four decades (Zhang et al., 2017). Oligotrophic waterbodies such as lakes and ponds are among the most threatened habitats in Europe and they harbor many endangered plant and animal species. However, changes in land use, nutrient inputs, and water pollution represent the main causes of the reduction in species diversity and abundance (Jamin et al., 2020). The dramatic decline in the number of such wetlands and resulting fragmentation is putting further pressure on their flora and fauna, thus these habitats have been protected by European law (Habitats Directive, 1992). In particular, mainly due to eutrophication, aquatic macrophytes belong to the most endangered groups (Preston and Croft, 2001). Anthropogenic increases in nutrients allow plant species adapted to this condition to grow faster and displace rare pioneer species (Kozlowski et al., 2009). Indeed, wetlands are rarely managed to protect threatened species, and it remains incompletely understood which conservation measures are likely to stabilize populations of endangered aquatic plants (Doust and Doust, 1995).

**Figure 1:**
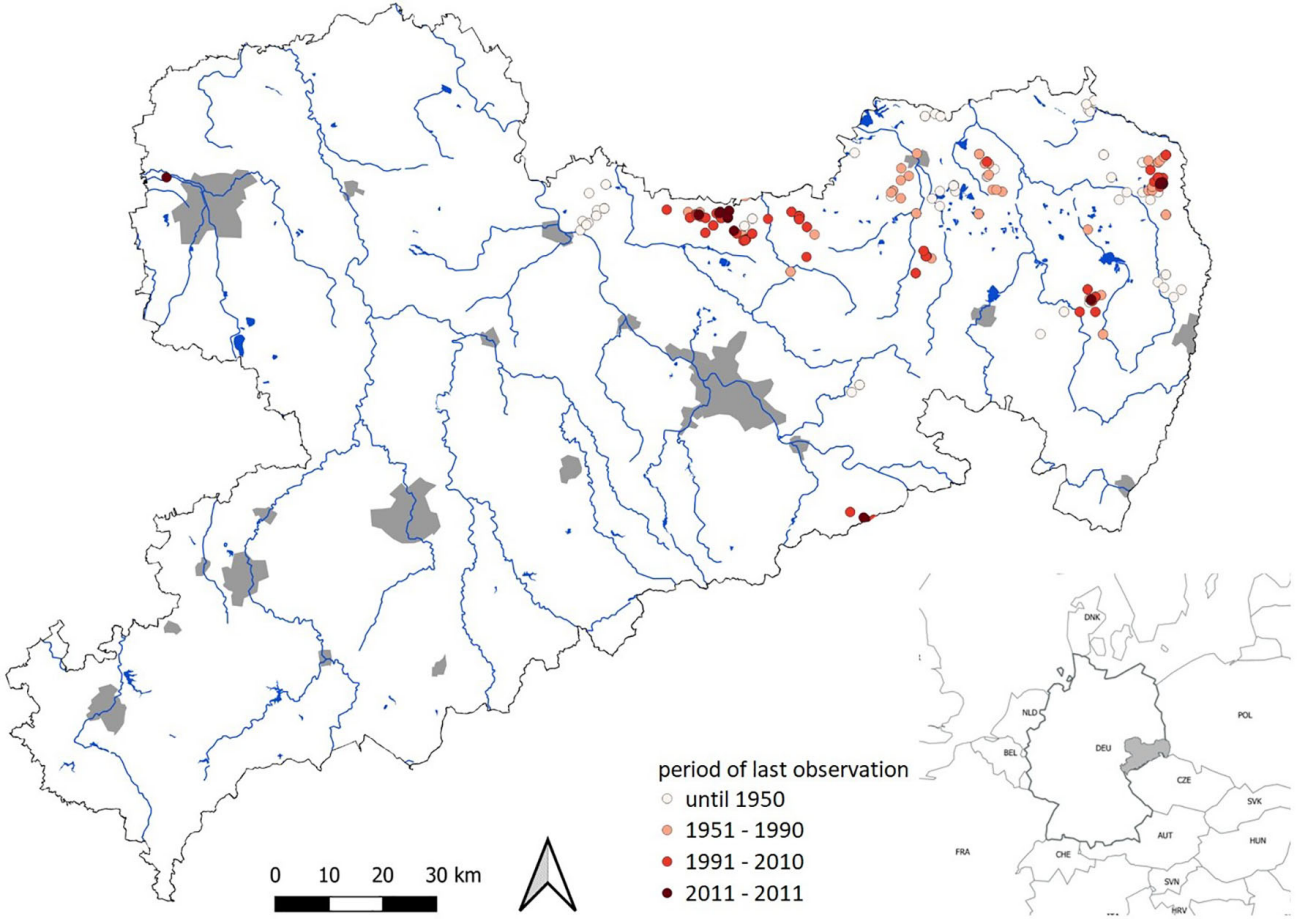
Occurrences of *Luronium natans* in Saxony (Germany) based on records from herbaria in Dresden (DR), Görlitz (GLM), Halle (HAL), Jena (JE) and Leipzig (LZ). Main rivers and water reservoirs are marked in blue, cities in grey.

*Luronium natans* (monotypic genus within Alismataceae) is a typical example of such an aquatic pioneer plant (Greulich et al., 2000; Szańkowski and Kłosowski, 2001) because it can hardly compete with more nitrophilic aquatic and semi-terrestrial plants (Szmeja, 2004). Throughout its distribution area covering the Atlantic and sub-Atlantic climatic zones of western and northwestern Europe, population trends are ‘declining’ (Preston and Hill, 1997; Greulich, 1999; IUCN, 2021). In Germany, *L. natans* is found mainly in northern lowlands. Since many populations have been lost since 1950, it has been classified as ‘critically endangered’ and became even extinct in some parts of Germany (Metzing et al., 2018). In the German Federal state Saxony, *L. natans* has suffered a significant population decline during recent decades (Schulz, 2013). Today, only a few populations remain in large, mostly extensively managed ponds in eastern Saxony (Hanspach, 2007), and botanical surveys report a continuous decline in populations (**Figure 1**). Although historical distribution is not fully documented, it is very likely that isolated populations have existed for the last 100 years. Population declines until 1990 are probably due to loss of suitable habitats, while more recent declines from 1990 onwards are not fully understood and may be caused by changes in water chemistry.

*Luronium natans* is a perennial herb hibernating by submerged rosettes adapted to both aquatic and semi-terrestrial environments with changing water levels (Szmeja, 2004). The species grows on muddy, usually peaty substrates and is part of the endangered oligotrophic standing water plant communities (Littorelletea uniflorae Br.-Bl. et R. Tx.; Greulich et al., 2000; Reißmann and Dieter, 2015). *Luronium natans* is morphologically highly variable because it develops distinct phenotypes adapted to both aquatic and semi-terrestrial environments (Kay et al., 1999; Szmeja, 2004). These phenotypic adaptations are highly plastic, may occur several times during an individual’s life time and these transformations need usually a few weeks only (Glück, 1905; Kay et al., 1999). The aquatic form grows in waters up to about 3 m in depth and is characterized by rosettes of linear submerged leaves and bundled roots. Individuals growing in shallower waters of up to 1.5 m depth may additionally develop floating leaves consisting of long petioles and leathery elliptical to oval blades (Szmeja, 2004). The semi-terrestrial form has well-developed, thread-like, mostly unbranched fine roots and its leaves shape resembles that of floating leaves, however, petioles are wider and shorter (Glück, 1905).

In addition to insect-pollinated flowers above the water level, plants can develop submerged cleistogamous flowers and reproduce vegetatively by stolons and fragmentation (Kay et al., 1999; Szmeja, 2004). However, the relative importance of these reproductive modes has been controversially discussed (Kay et al., 1999; Nielsen et al., 2006; Cox et al., 2014). Nuts do not have special adaptations, but both dispersal by water or birds seems to be likely (Kay et al., 1999; Halvorsen and Grostad, 2002). The species forms a considerable seed bank with seeds germinating after long periods of stasis at considerable rates (Glück, 1905; Nielsen et al., 2006) and the seedbank is considered to play a key role for surviving periods of inadequate conditions (Kaplan et al., 2014). Particularly in clonal populations, seeds are the major carrier of long-distance spread (Eckert et al., 2016).

Because sufficient genetic variation is the basis for the adaptability to the ever-changing environment (Hoban et al., 2020) and thus for the survival of a species (Hughes et al., 1997; Luck et al., 2003), assessing the extent of genetic diversity in endangered plant species has become a fundamental tool for conservation efforts. Still, the role of genetic diversity for conservation is often under-appreciated in plants and explicit goals for genetic diversity are undeveloped or focus mostly on species of agricultural relevance (Hoban et al., 2020). The importance of intraspecific genetic diversity for the resilience of ecosystems and survival of species is crucial and confirmed by a large body scientific evidence (Laikre et al., 2020). However, estimation of the effective population size (Ne) in natural plant populations with a polymorphic reproductive strategy and a considerable seed bank is not straight-forward at least using easily available dominant marker systems, so most studies rely on simple estimators of genetic diversity (Allendorf et al., 2013). Moreover, dramatic habitat losses due to land use changes may happen so rapidly that they are often not mirrored by lagging changes in genetic diversity (Reichel et al., 2016; Aavik et al., 2019).

*Ex-situ* collections can be effective measures in conserving plant species (Abeli et al., 2020) by preventing extinction and restoring a species’ historic range by re-introductions (He et al., 2016). Therefore, the knowledge of the genetic status and relatedness of source populations can significantly influence re-introduction efforts by maximizing genetic variation in target populations (He et al., 2016; Robinson et al., 2021).

As with many other endangered aquatic plants, neither population genetics nor ecology of *L. natans* has been intensively studied and data are rather anecdotal across its distribution area. So far, population genetic studies have been carried out in the Czech Republic, Germany, Wales and Ireland using isozymes (Kay et al., 1999; Bartuška, 2009) as well as in Belgium and Denmark using Amplified Fragment Length Polymorphism (AFLP; Nielsen et al., 2006; Cox et al., 2014). Bartuška (2009) found little genetic diversity within populations from the Czech Republic and Germany (Saxony: *Großenhainer Pflege* and *Niederspree*). Furthermore, Belgian populations were found to have a high degree of clonal reproduction (Cox et al., 2014).

However, effective reintroduction requires knowledge not only of the genetic status of populations, but ecological experiments on plant growth and survival, which is particularly important for a species with diverse growth forms such as *Luronium*.

To get fundamental genetic and ecological knowledge for successful re-introduction efforts of *Luronium natans*, we investigated population genetics of the remaining Saxon populations using AFLPs. Although this method relies on presence/absence data of anonymous fragments and may have disadvantages compared to sequence-based population genomic methods it still provides reliable and cost-effective data for population genetics in species without prior knowledge on the genome (Woodhead et al., 2005; Allendorf et al., 2013). These markers yield a genome-wide overview about population genetic patterns and robust data can be retrieved when a rigorous scoring and replication scheme is applied (Ley and Hardy, 2013, see below). In addition, previous studies on other European populations of *Luronium natans* were also performed with AFLPs (Cox et al., 2014; Nielsen et al., 2006), allowing for direct comparisons of population genetic estimators.

In particular, we determined the genetic diversity within populations to estimate the degree of clonality and thus the influence of sexual versus vegetative reproduction. We also studied the genetic distance of these populations in comparison to samples covering the species’ distribution area and with material from *ex-situ* cultures from various botanical gardens. Since all previous publications indicated a low genetic variability within populations in *L. natans* (Kay et al., 1999; Nielsen et al., 2006; Bartuška, 2009; Cox et al., 2014) and such a pattern seems to hold in general for aquatic plants (Santamaría, 2002) because clonal propagation appears to be common in endangered and aquatic species populations (Silvertown, 2008), we hypothesize low levels of genetic diversity within Saxonian populations but a considerable genetic variance between them.

In addition, we conducted a greenhouse experiment to investigate the influence of temperature, substrate, and water level on plant growth in order to find optimum growing conditions for *ex-situ* cultures potentially used for re-introduction experiments.

## 2 Material and methods

### 2.1 Study sites

Our sampling of *L. natans* focused on Saxony (Germany), where the species is found in two pond areas approximately 90 km distant from each other: *Niederspree* (DE_Ni) and *Großenhainer Pflege* (DE_Gr; Hanspach, 2007; Hanspach et al, 2016). The occurrences in the *Niederspree* pond area have been known at least since 1899 and in *Großenhainer Pflege* since 1840.

Both pond areas contain a number of distinct populations in the respective single ponds (**Table 1**). Within the pond areas several thousand shoots have been detected, in which high fluctuation rates up to 50% were observed (Hanspach et al., 2016, own observations). However, despite historical records (personal communication L. Runge) no occurrences of *Luronium* were found at *Goldgrubenteiche*. The material was collected from the end of September to the beginning of October 2020. In accordance with the collection permit issued by the relevant authorities leaves from 1−28 plants per pound (depending on the size of the subpopulation; minimum distance between samples – 0.8 m) were collected and dried in silica gel. To ensure that the harvested material came from one plant, only a single leaf per plant was harvested. In many cases plants occurred in rather dense patches, from which we sampled only one leaf to avoid collecting clonal plants.

**Table 1:** Origin of sampled material.

country, state	locality, population	subpopulation	GPS coordinates (WGS 84)		No of samples
Germany, Saxony	*Großenhainer Pflege* (DE_Gr), *Tiergartenteich* (DE_Gr_Tier)	large *Tiergartenteich* (TierL); additional sample from Botanical Garden BG_2: (accession number: 017947-28)	51.338 N	13.746 E	23 1
		small *Tiergartenteich* (TierS); additional sample from Botanical Garden BG_1: (accession number: 017947-28)	51.339 N	13.746 E	8 1
		*Schwarzteich^1^*; (Sch)			1
	*Großenhainer Pflege* (DE_Gr), *Raschützwaldteich* (DE_Gr_Ras)	*Kleiner Teich* near *Raschützwald*, eastern part (RasE)	51.342 N	13.670 E	28
		*Kleiner Teich* near *Raschützwald*, western part (RasW)	51.342 N	13.668 E	13
	*Großenhainer Pflege* (DE_Gr), *Sergkteich* (DE_Gr_Ser)	southern *Sergkteich* (SerS)	51.345 N	13.724 E	12
		northern *Sergkteich* (SerN)	51.348 N	13.726 E	2
Germany, Saxony	pond area *Niederspree* (DE_Ni), *Großer Tiefzug* (DE_Ni_Tief)	eastern part (TiefE_1)	51.403 N	14.905 E	19
		eastern part, ditch (TiefE_2)	51.403 N	14.905 E	1
	pond area *Niederspree* (DE_Ni), *Froschteich* (DE_Ni_Fro)	*Froschteich* (Fro); additional sample from Botanical Garden BG_4 (accession number: 018092-20)	51.404 N	14.908 E	3 1
Germany, Bavaria	Bad Alexandersbad (DE_By)		50.833 N	14.155 E	6
Germany, Mecklenburg-Pomerania	small patch near to *Entensee* and *Schwarzer See* (DE_Ro)		50.832 N	14.202 E	12
Czech Republic, Ustecky Kraj	Rybník u Králova mlýna (CZ_1)		50.833 N	14.155 E	6
	Hasičská nádrž (CZ_2)		50.832 N	14.202 E	12
	Pruhonice Park (Czech Academy of Sciences) (BG_3)		ex cult., unknown		3
Norway, Oslo	Lake Breisjøen (NO_1)		50.833 N	14.155 E	6
	Lake Maridalsvannet (NO_2)		50.832 N	14.202 E	12
Poland, Pomeranian	Jezioro Smołowskie (PL)		50.833 N	14.155 E	6
United Kingdom, North Wales	Llyn Padarn (UK)		50.832 N	14.202 E	12

^1^Population was newly established based on material from *Tiergartenteich* in 2019 (pers. comm. L. Runge, head of the regional association ‘*Großenhainer Pflege*’ of non-governental organization ‘*Naturschutzbund Deutschland’*).

Additionally, leaves from plants of two other localities in Germany (Mecklenburg-Vorpommern: near Rostock; Bavaria: Bad Alexandersbad), as well as several samples from the Czech Republic, Poland, Great Britain, and Norway were investigated (**Table 1**). Furthermore, leaves from plants cultivated in the Botanical Garden of the TU Dresden were also studied (**Table 1**).

### 2.2 Population genetic analyses

DNA was isolated from silica gel dried leaf material using innuPREP Plant DNA Kit (Analytic Jena, Germany) following the manufacturer’s instructions except for the elution of DNA, which was eluted in two steps: 1. with 70 µl HPLC grade water, 2. with 30 µl HPLC grade water. The quality of isolates was checked with agarose gel electrophoresis (1%) and DNA quantity was estimated using Qubit Fluorometer (Thermo Fisher Scientific). Extracted DNA was stored at -22°C until further processing.

To investigate genetic diversity within Saxon populations and their relationships to other European populations, AFLP (Vos et al., 1995) was used with minor modifications. One hundred nanograms of isolated DNA were digested with the restriction enzymes *PstI* and *MseI*. Restriction and ligation were performed in a single reaction for 10 hours at 37°C. For selective PCR, we followed Schuelke (2000). We first tested 15 primer combinations based on previous AFLP studies on *L. natans* (Cox et al., 2014; Nielsen et al., 2006) and selected four combinations for further analyses: S7 (*PstI*-ACG/*MseI*-CAC), S8 (*PstI*-ACG/*MseI*-CCG), S11 (*PstI*-ACT/*MseI*-CTC), S12 (*PstI*-ACT/*MseI*-GCT). Samples were randomly distributed on 96-well plates to avoid position effects. Sixty-six of 253 samples were analyzed twice for quality control. In addition, five identical samples were repeated on each of the five 96-well plates, and negative controls for restriction/ligation, pre-selective PCR, and selective PCR were included. Automated detection of AFLP fragments was performed by the Senckenberg Biodiversity and Climate Research Center (SBik-F; Frankfurt am Main, Germany) with an ABI 3730 sequencer (ABI Life Technologies, Darmstadt, Germany) using the LIZ-600 size standard (ABI Life Technologies).

Fragment scoring was processed as described in Ley and Hardy (2013). First, scoring was automatically done using PeakScanner 1.0 (Applied Biosystems, ThermoFisher Scientific, Berlin, Germany) on a size range from 100–500 bp, the minimal peak height of 30, and maximal peak width of 1. The program TinyFLP v1.30 (Arthofer, 2010) was then used for a pre-choice of markers. Finally, the software SPAGeDi (Hardy and Vekemans, 2002) was used to estimate the reproducibility of bands by calculating fragment-wise Fst values across repeated samples by combining all combinations within one matrix (see details in Ley and Hardy, 2013). We retained a final data matrix consisting of 151 samples from 22 (sub)populations with 48 fragments with a Fst ≥0.25.

To estimate the genetic diversity within populations (for those with >5 samples) we calculated Expected Heterozygosity (He); Shannon’s Index of Diversity (I), and the Percentage of Polymorphic Loci (%P) with GenAIEx v. 6.5 (Peakall and Smouse, 2012). Population structure was analyzed using GenAIEx by performing Analysis of Molecular Variance (AMOVA) and Principal Coordinate Analysis (PCoA) based on Jaccard distances with the R-Package vegan (Oksanen et al., 2020). In addition, we performed Mantel tests (Mantel, 1967) with GenAIEx to check for a correlation between geographic and genetic distances. Furthermore, Bayesian clustering was conducted with the R-package ParallelStructure (Besnier and Glover, 2013) with 10 iterations for every K from 1 to 10, with a burn-in of 500,000 generations followed by 1,000,000 generations. The best-fitting model was chosen according to the method described by Evanno et al. (2005) using the software Structure Harvester v.0.6.94 (Earl and vonHoldt, 2012). Results were visualized with the program DISTRUCT v.1.1 (Rosenberg, 2003).

### 2.3 Experiment on plant growth

The experiment started on October 27, 2020 and ended on March 12, 2021. Plant material for the experiment originated from the population *Kleiner Tiergartenteich* (DE_Gr_Tier; **Table 1**) and had been cultivated since 2019 in the Botanical Garden of the TU Dresden. The plants used for the experiment were propagated by runners from mother plants and were grown after separation from the mother plants for 7−8 weeks in submerged pots in the outside area of the botanical garden.

Conditions tested in this experiment were selected based on observations from habitat and previous publications (Glück, 1905; Barrat-Segretain and Bornette, 2000; Nielsen et al., 2006). Plants were potted into four types of substrates: clay, sand, mixed, i.e. clay/sand mixture (1:1), and layered, i.e. sand as the top layer and clay as the bottom layer (1:1) to check whether lack of nutrients (especially sand as top layer) will influence root length. Pots were subsequently placed in three different water levels: (1) semi-terrestrial condition (saturated): water up to about 3 cm below the rim of the pot; (2) aquatic condition I: water about 0.5 – 1.0 cm above the rim of the pot; (3) aquatic condition II: water about 7 cm above the rim of the pot. Since seasonal differences in plant growth have been observed previously which are likely related to water temperature (Barrat-Segretain and Bornette, 2000; Nielsen et al., 2006), plants were exposed to two different water temperatures: cold: ~ 5 °C and warm: ~ 14 °C. Four plants each, i.e. biological replications were used for each combination of substrate, water level, and temperature (**Supplementary Figure 1**), thus the experiment constituted a full-factorial design. Water level (rainwater) was regularly checked and replenished when necessary. The plants were illuminated with assimilation lighting from 7 to 10 a.m. Since not all plants used for the experiments were of the same size, their size classes were recorded in the beginning of the experiment: small (s), medium without floating leaves (m), and medium with floating leaves (wfl). After completion of the experiment roots of each plant were rinsed with water to remove remaining substrate. Then, the length of the longest root and of the longest leaf (floating leaves were excluded) were measured. Typically, roots are cut and weighted, but this procedure could not be performed since the plants had to be kept alive for further *ex situ* conservation.

The effects of the substrate, water level, and temperature on root and leaf length were analyzed using linear regressions. Additionally, we analyzed the effect of the plant size at the onset of the experiment by including the variable as a covariate. For each response variable, we calculated 27 different models (**Supplementary Tables 1**, **3**) with the predictor variables in plausible combinations. For model selection, we used Akaike’s information criterion (AIC). The 27 models were ranked *via* AIC and the model with the lowest AIC and a difference of at least 2 to the next model was then considered as the best-fitting model (Burnham and Anderson, 2002). All analyses were performed using the R environment (R Core Team, 2020).

## 3 Results

### 3.1 Genetic diversity of *Luronium* populations

Genetic diversity of *L. natans* populations was reasonably high **Table 2**; (grand means He = 0.25; Shannon-Index = 0.387) and differed only little between populations, with highest values found in the population *Raschützteich* (DE_Gr_Ras: He = 0.268) and lowest diversity detected in the Norwegian population (NO: He = 0.234).

**Table 2:** Overview about estimators of genetic diversity in populations >5 samples.

Country	Population ID	N	Shannon-Index	He	%P
Czech Republic	CZ	17	0.379 ± 0.033	0.243 ± 0.024	83.3%
Norway	NO	9	0.367 ± 0.033	0.234 ± 0.024	83.3%
Germany (Saxony)	DE_Ni	21	0.370 ± 0.036	0.240 ± 0.026	81.3%
	DE_Gr_Ras	41	0.416 ± 0.031	0.268 ± 0.023	95.8%
	DE_Gr_Ser	13	0.392 ± 0.035	0.256 ± 0.026	83.3%
	DE_Gr_Tier	31	0.398 ± 0.032	0.256 ± 0.024	93.8%
**Total**		**132**	**0.387 ± 0.014**	**0.250 ± 0.010**	**86.8% ± 2.6%**

Abbreviations for populations are according to Table 1. He, Expected Heterozygosity, %P, Percentage of Polymorphic Loci. Values in bold are sums or mean values (total) of the above values.

### 3.2 Genetic structure of populations

**Figure 2:**
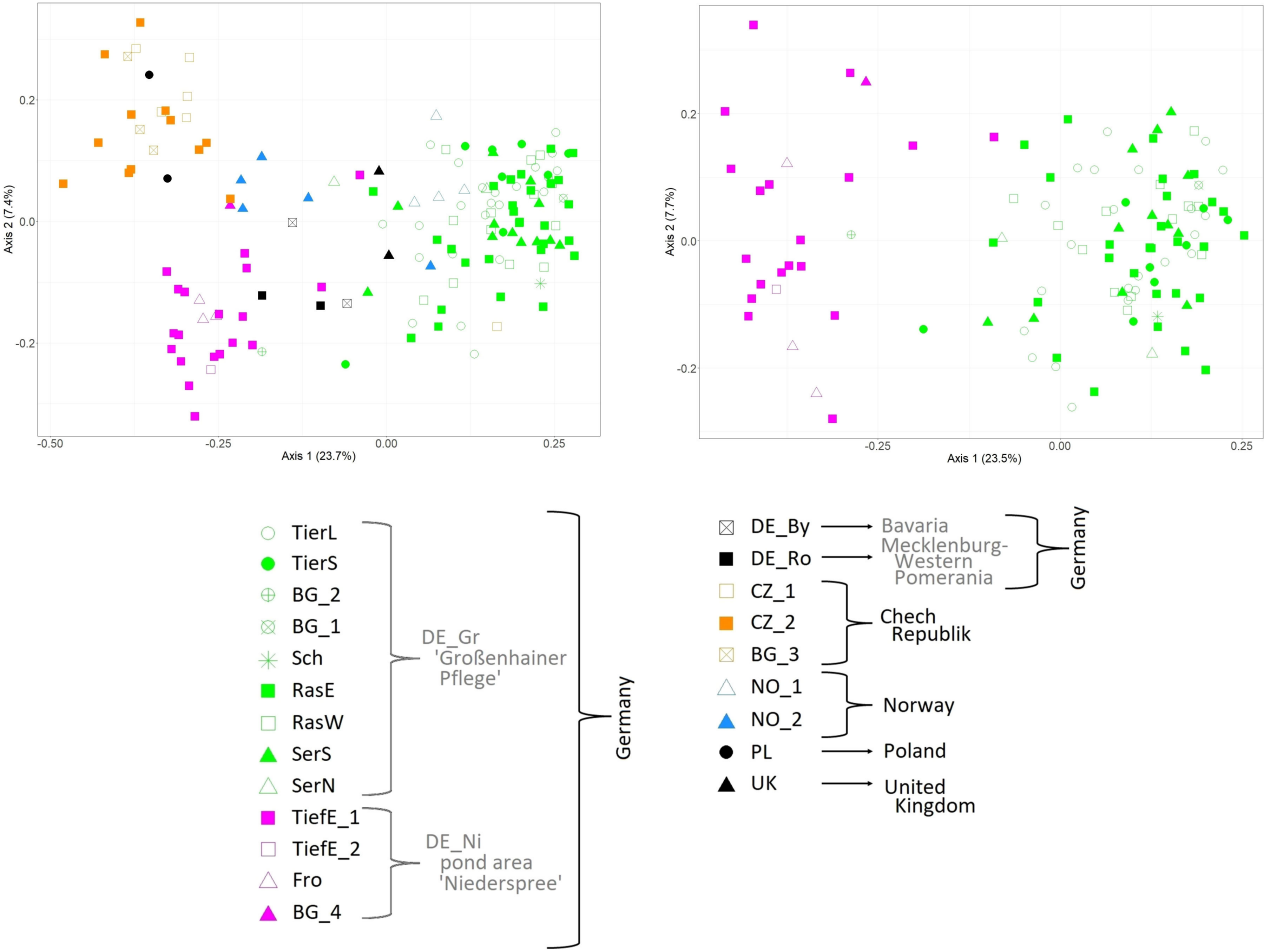
Principal Coordinate Analysis based on 48 AFLP loci of 151 individuals from European *L. natans* populations (left), and 113 individuals from Saxon populations only (right). Abbreviations for populations are according to **Table 1**.

The Principal Coordinate Analyses including all 151 samples across Europe separated mainly samples from Eastern Europe (Czech Republic = CZ and Poland =PL) and those from Eastern Saxony (pond area *Niederspree* = DE_Ni) from the remaining samples along the first axis (**Figure 2**). In the left part of the plot samples from the Central Saxon area *Großenhainer Pflege* (DE_Gr) were clustered, whereas samples from Northern and Southern Germany (DE_Ro, DE_By), Great Britain (UK), and Norway (NO) were found in a rather intermediate position. Note that samples obtained from *ex situ* collections of Botanical Gardens clustered according to their geographical origin (**Table 1**). The PCoA presented in **Figure 2** was based on Saxon samples only. Accordingly, samples from *Niederspree* (Eastern Saxony) were clearly separated along the first axis. We observed no genetic structure among the populations within both pond areas, respectively.

**Figure 3:**
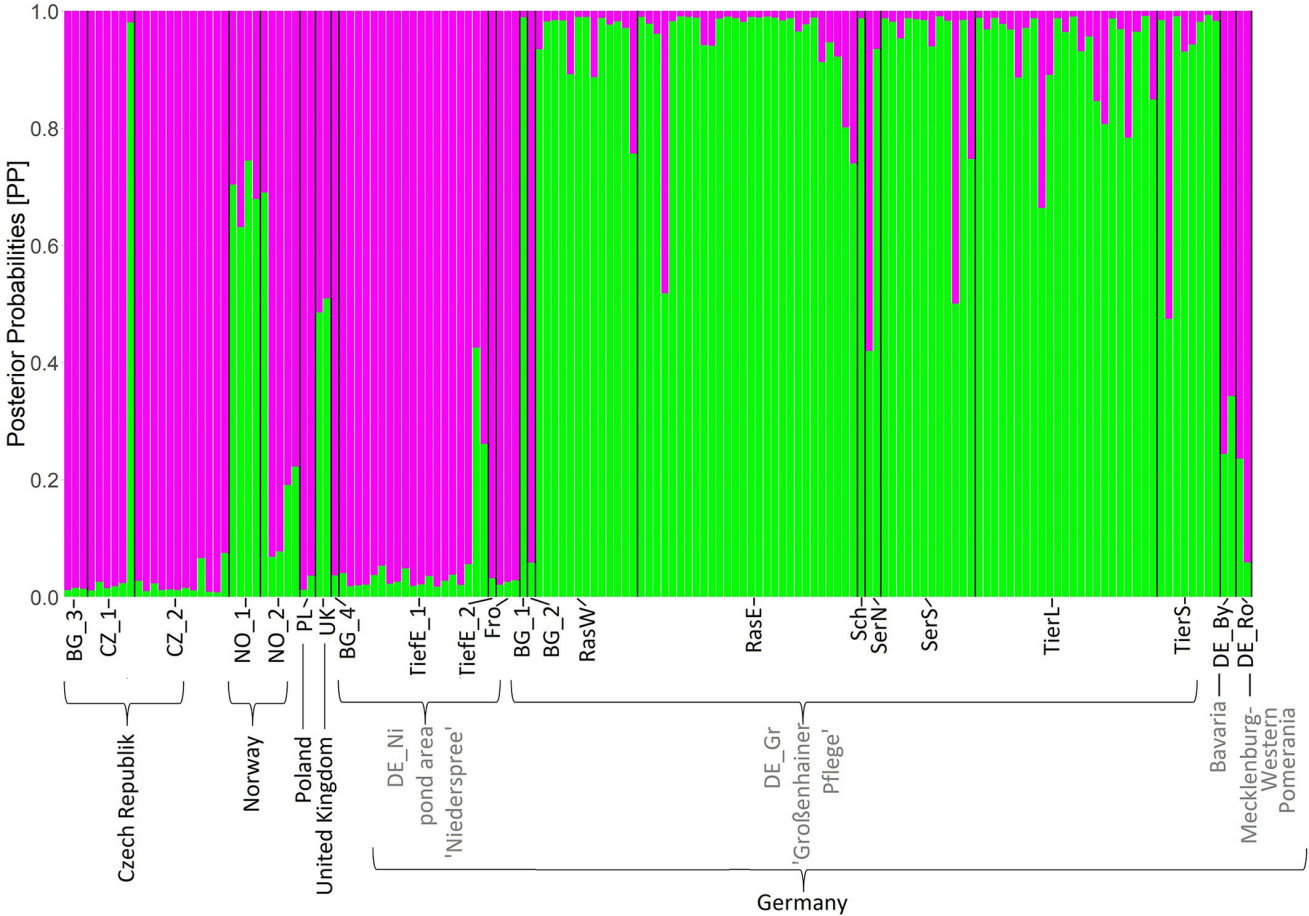
Bayesian clustering based on 48 loci AFLP for the model K = 2. Length of the colored bars indicates posterior probabilities (PP) for belonging to one of the two clusters (pink and green). Abbreviations for populations are according to **Table 1**.

Analyses of Molecular Variance (**Table 3**) revealed a moderate differentiation (ΦPT = 0.334, P ≥ 0.001) between both Saxon localities (DE_Gr and DE_Ni). Results from Bayesian clustering (**Figure 3**) with two clusters (K = 2) roughly corresponded to the PCoA analyses of all samples. Accessions from the area *Großenhainer Pflege* (DE_Gr) were assigned to the green cluster, whereas populations from *Niederspree* were found in the same cluster containing most of the Czech (CZ), Polish (PL), and Norwegian (NO) samples. Admixture between both clusters was detected for a few samples from various ponds in Central Saxony and for plants originating from Norway (NO) and Wales (UK). Mantel test did not detect a significant correlation between geographic and genetic distances (all populations: R^2^ = 0.0271; p = 0.100, Saxon populations: R^2^ = 0.9974 and p = 0.078).

**Table 3:** Analysis of Molecular Variance based on 48 AFLP loci among and within two Saxon localities of *Luronium natans* (*Niederspree*: DE_Ni and *Großenhain*: DE_Gr).

Source of Variation	Df	Sum of Squares	Mean of Squares	Estimated Variance	% Variance
Among localities	3	122.791	122.791	3.442	33%
Within localities	103	712.511	6.851	6.851	67%
Total	106	835.302		10.294	100%

df, degrees of freedom.

### 3.3 Plant growth

**Figure 4:**
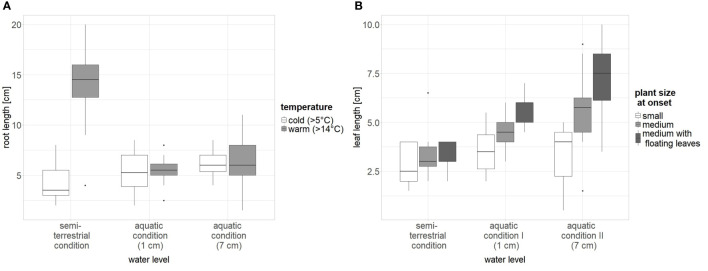
The influence of significant factors on plant growth (**A**: root length and **B**: leaf length) of *L. natans*. Water level: “1 cm”: water about 0.5 − 1 cm above the rim of the pot; “7 cm”: water about 7 cm above the rim of the pot; saturated: semi-terrestrial form, water only up to about 3 cm below the rim of the pot; temperature: cold ~ 5°C, warm ~ 14°C.

The best-fitting model for root length (adjusted r-squared 0.65, p < 0.001) included an interaction between temperature and water level (**Supplementary Tables 1**, **2**). The size of the plants at the beginning of the experiment did not have remarkable effects on root length. Roots were longer in plants grown under high water levels than under low water levels. The effect of temperature on the root length was restricted to plants grown under saturated water conditions: here, warm temperature led to roots three times longer than those of plants grown under cold temperatures (**Figure 4**).

The best-fitting model for leaf length (adjusted r-squared 0.47, p < 0.001) included an interaction between plant size at the onset of the experiment and water level. According to the best-fitting model (**Supplementary Tables 3**, **4**), leaf length increased with the water level. Plants of medium size and with already developed floating leaves had the longest leaves, and this effect tended to increase with increasing water depths (**Figure 4**). However, the selected model did not fit much better than the model with plant size at onset and water level lacking interaction, and the model including plant size at onset, water level, and temperature (Δ AIC of the first three models was <2).

## 4 Discussion

### 4.1 Genetic diversity in *L. natans* populations

In contrast to our initial hypothesis of low genetic variation within populations, the values of the indices of genetic diversity were quite high (I = 0.367−0.416; **Table 2**), and these results suggest that Saxon populations are not genetically impoverished and that clonal reproduction does not cover the effect of the sexual reproduction. This finding was rather unexpected and contradicts the results from Bartušek (2009), who reported a moderate to very low (sometimes absent) genetic diversity in Czech and Saxon populations but applied possibly less polymorphic isozyme markers compared to AFLPs (Ipek et al., 2003; Mondini et al., 2009). Similarly, Kay et al. (1999) suggested the dominance of clonal propagation in the United Kingdom and Ireland based on isozyme analyses, and also previous studies from Belgium reported considerably low values (I = 0.0996) based on AFLPs (Cox et al., 2014). In addition, Szmeja (2004) assumed that *L. natans* populations in Poland are mainly represented by multigenerational clones reproducing primarily by fragmentation. However, he observed plants in water reservoirs with deeper water bodies, where cleistogamy occurs more often (Kay et al., 1999). Thus, the high genetic diversity observed in the Saxon populations may be due to marked outcrossing from insect-pollinated chasmogamic flowers above the water surface, which we observed in all ponds studied. Moreover, *L. natans* produces a substantial seed bank and is characterized by high rates of seed germination (average 51-60%; Nielsen et al., 2006). Lansdown and Wade (2003) assumed the seeds to have a capacity for extended dormancy over many years. Germination might take place preferably in shallow waters or on naked mud in empty ponds. The extreme fluctuations of observed shoots (up to 50%) between years (Hanspach et al., 2016, own observations) can be caused by pronounced clonal growth or by high proportion of regeneration from seeds. A much denser sampling design in consecutive years may help to investigate the different ways of population growth. Moreover, estimators of genetic diversity respond often delayed compared to rapid changes in habitats and thus in strong declines in population size (Reichel et al., 2016; Aavik et al., 2019).

In contrast to our results, low values of genetic diversity based on AFLPs (I = 0.025−0.140) were reported within three species of the closely related genus *Baldellia* (Alismataceae) in Europe (Arrigo et al., 2011). However, these authors found varying values depending on the geographic origin of the samples with higher values detected at Iberian Peninsula compared to France and Switzerland, which were explained by a recent post-glacial re-colonization to the North. Thus, considering a species’ biogeographic history might also be crucial regarding conservation approaches.

### 4.2 Genetic structure of *L. natans* populations

According to our hypothesis of a pronounced genetic structure between Saxon populations, *L. natans* samples clustered mainly according to their geographic origin, whereby we detected three groups of samples: (1) Czech populations, (2) Saxon populations from *Niederspree* and (3) Saxon populations from the pond area *Großenhainer Pflege*, with remaining samples from Germany and Norway in between (**Figure 2**). This partially coincides with the results reported by Bartuška (2009), who showed that the populations from the Czech Republic, and the two Saxon populations *Großenhainer Pflege* and *Niederspree* constituted three separate groups. Remarkably, sites from Saxony, which are only separated by a distance of approximately 90 km appeared to be rather distantly related because samples from the area *Großenhainer Pflege* formed a separate cluster and those from *Niederspree* were close to remaining samples from various sites across Europe (**Figure 3**). Most accessions were assigned with high posterior probabilities to one of the two clusters (**Figure 3**), thus admixture between both Saxon areas played a minor role. Given the rather low geographic distance between Saxon sites the ΦPT value of 0.334 suggests a moderate level of genetic differentiation between populations (**Table 3**), which was comparable to the values observed in Belgian populations ranging between 0.226 and 0.455 (Cox et al., 2014). Samples from neighboring ponds within each of the respective Saxon areas appeared to be completely intermingled, thus, no genetic structuring on very short distances was observed (**Figure 2**). While the lack of genetic structure between sub-populations per population (**Table 1**) may be an effect of water-mediated dispersal (Kay et al., 1999), it does not explain the situation between the three populations in *Großenhainer Pflege* (DE_Gr_Tier, DE_Gr_Ras, and DE_Gr_Ser) as these areas are not connected by ditches but rather suggests zoochoric dispersal, e.g. by birds. Although, Mikkelsen (1943) argued that *Luronium* nuts lack adaptations to bird dispersal, more recent studies reported likely dispersals of 50 to 100 km (Fritz, 1989; Halvorsen and Grostad, 2002; Lansdown and Wade, 2003; Nielsen et al., 2006). Here, bird dispersal would only explain the genetic similarity at the local scale (**Table 1**) but not between areas, because both areas in Saxony (DE_Gr and DE_Ni) were clearly separated (**Figures 2**, **3**). However, we detected no significant pattern of isolation by distance (Mantel tests for European populations: R^2^ = 0.0271; P = 0.100, for Saxony R^2^ = 0.9974 and P = 0.078) but due to the restricted availability of plant material our sampling was rather unbalanced.

Remarkably the genetic similarity of Polish (PL) and Czech (CZ) samples (**Figure 2**) is surprising, given the geographic distance between the populations. We would have rather expected a close relationship of the Polish samples with the German population from Rostock (DE_Ro). In Poland, two big geographically separated occurrences, i.e. Pomerania (current populations; closer to Rostock) and Lower Silesia (historical populations; closer to the Czech Republic) are present (Dajdok and Proćków, 2003; Szmeja, 2004). Our findings are in line with the observation of Nischkowsky and Schube (1908) and Suda et al. (2000), who referred to the Lower Silesian occurrences as part of the Jizera Foothills meta-population, which also include the oldest Czech populations. The Czech populations examined in the present study are about 120 km away from these historical occurrences (Nischkowsky and Schube, 1908) but may represent together with scattered populations from *Wielkopolska*, Poland (Szmeja, 2007; Szmeja, 2013) remnants of a former widespread distribution in Poland and the Czech Republic. The closer relationship of samples from *Niederspree* (DE_Ni) to Czech populations rather than to the more western Saxon samples from *Großenhainer Pflege* (DE_Gr) implies a geographic separation of *L. natans* between Eastern and Western Europe, whose border might cross Saxony.

### 4.3 Growing conditions of *L. natans*

Our experiment showed that *Luronium* plants developed longer roots under semi-terrestrial (saturated) conditions at higher temperature (**Figure 4**). This is in line with the assumption of Glück (1905) who argued that a more extensive root system of semi-terrestrial forms may be caused by their restricted access to water. The here observed longer roots at higher temperatures fit also with *Luronium’s* Ellenberg Indicator Value for temperature (T6; Ellenberg et al., 2001) indicating its adaptation to moderate warm to warm temperatures. In addition, we found longer leaves in plants growing at higher water level, which can be explained by the fact that the aquatic form of *Luronium* has longer leaves than the semi-terrestrial form. In addition, plants which were larger at the onset also produced longer leaves, however, plant size at onset was an uncontrolled factor, and the plants were randomly selected according to size. Thus, the best results for obtaining robust plants for potential reintroduction are obtained under semi-terrestrial conditions and at higher temperatures, as a strong root system can facilitate active planting.

### 4.4 Implications for conservation

Our study revealed moderate levels of genetic diversities in Saxon populations suggesting that the observed decline in populations is not caused by lacking genetic diversity. We assume that populations in ponds rely strongly on regeneration by seeds emerged from outcrossing rather than by clonal reproduction or cleistogamous flowers. While we found a considerable genetic differentiation among Saxon populations our results showed no genetic differentiation within pond systems indicating sufficient genetic exchange between neighboring ponds probably mediated by birds or water. Thus, our results imply to use rather nearby populations from the same area as source for reintroductions. Our plant growth experiments demonstrated that possible reintroductions of plants are facilitated by optimal growing conditions in gardens since we obtained plants with longest roots when grown under saturated water conditions and higher temperatures, whereas the tested soil type seems to have less impact.

## Data availability statement

The raw data supporting the conclusions of this article will be made available by the authors, without undue reservation.

## Author contributions

CR, SW, BD, and FR planned the study, WM and BD collected the samples, WM did the laboratory work, WM, VH, and CR performed population genetic analyses, JA performed statistical analyses on plant growth, WM, JA, CR, and SW wrote the manuscript. All authors read, contributed and approved the final manuscript.
