# Supplementary material for: Population genetics and plant growth experiments as prerequisite for conservation measures of the rare European aquatic plant Luronium natans (Alismataceae)
Source: Front Plant Sci. 2023 Jan 13;13:1069842. doi: 10.3389/fpls.2022.1069842 (PMC9880460; doi:10.3389/fpls.2022.1069842)
Supplement: Supplementary file 1 [file DataSheet_1.docx]

***Supplementary Material***

1. **Supplementary Figures**

**Supplementary Figure 1.** Compilation of the combinations of treatments used for the root growth experiment.

1. **Supplementary Tables**

**Supplementary Table 1** Results of root length model selection. AIC - Akaike’s information criterion.

| **model nr.** | **predictors** | **df** | **AIC** | **deltaAIC** |
| --- | --- | --- | --- | --- |
| m11 | water * temperature | 7 | 434.6039 | 0 |
| m12 | substrate * water * temperature | 25 | 446.0931 | 11.4892 |
| m26 | size at onset * water * temperature | 19 | 446.9097 | 12.3058 |
| m27 | size at onset * substrate * water * temperature | 52 | 459.8207 | 25.2168 |
| m7 | water + temperature | 5 | 493.4092 | 58.8053 |
| m19 | size at onset + water + temperature | 7 | 494.7945 | 60.1906 |
| m8 | substrate + water + temperature | 8 | 495.8322 | 61.2283 |
| m20 | size at onset + substrate + water + temperature | 10 | 496.7800 | 62.1761 |
| m15 | size at onset + water | 6 | 510.1435 | 75.5396 |
| m3 | water | 4 | 510.5162 | 75.9123 |
| m22 | size at onset * water | 10 | 512.3003 | 77.6964 |
| m17 | size at onset + substrate + water | 9 | 513.0785 | 78.4746 |
| m5 | substrate + water | 7 | 513.3449 | 78.7410 |
| m4 | temperature | 3 | 514.5071 | 79.9032 |
| m16 | size at onset + temperature | 5 | 517.1689 | 82.5650 |
| m6 | substrate + temperature | 6 | 518.1878 | 83.5839 |
| m23 | size at onset * temperature | 7 | 519.8002 | 85.1963 |
| m9 | substrate * water | 13 | 520.1717 | 85.5678 |
| m18 | size at onset + substrate + temperature | 8 | 520.3762 | 85.7723 |
| m25 | size at onset * substrate * temperature | 23 | 521.1996 | 86.5957 |
| m10 | substrate * temperature | 9 | 523.1804 | 88.5765 |
| m1 | NULL | 2 | 528.8895 | 94.2856 |
| m13 | size at onset | 4 | 531.2224 | 96.6185 |
| m2 | substrate | 5 | 532.8088 | 98.2049 |
| m14 | size at onset + substrate | 7 | 535.0828 | 100.4789 |
| m21 | size at onset * substrate | 13 | 538.6078 | 104.0039 |
| m24 | size at onset * substrate * water | 34 | 540.3874 | 105.7835 |

**Supplementary Table 2** Root length model test table for the model m11. Signif. Codes: 0 '***', 0.001 '**', 0.01 '*', 0.05 '.', 0.1 ''; Residual standard error: 2.343 on 88 degrees of freedom; Multiple R-squared: 0.6703, Adjusted R-squared: 0.6515; F-statistic: 35.77 on 5 and 88 degrees of freedom, p-value <2.20E-16

| **variable** | **estimate** | **std. error** | **t-value** | **p-value** | **sign.** |
| --- | --- | --- | --- | --- | --- |
| (Intercept) | 4.2692 | 0.6497 | 6.571 | 3.41E-09 | *** |
| water level 1 cm | 0.8870 | 0.8747 | 1.014 | 0.313 |  |
| water level 7 cm | 1.9808 | 0.8747 | 2.264 | 0.026 | * |
| temperature warm | 9.6683 | 0.8747 | 11.053 | 2.00E-16 | *** |
| water level 1 cm : temperature warm | -9.3558 | 1.2047 | -7.766 | 1.39E-11 | *** |
| water level 7 cm : temperature warm | -9.8300 | 1.1963 | -8.217 | 1.67E-12 | *** |

**Supplementary Table 3** Results of leaf length model selection. AIC - Akaike’s information criterion.

| **model nr.** | **predictors** | **df** | **AIC** | **delta_AIC** |
| --- | --- | --- | --- | --- |
| m22 | size at onset * water | 10 | 345.9287 | 0 |
| m15 | size at onset + water | 6 | 346.4536 | 0.5249 |
| m19 | size at onset + water + temperature | 7 | 347.1833 | 1.2546 |
| m17 | size at onset + substrate + water | 9 | 349.8420 | 3.9133 |
| m20 | size at onset + substrate + water + temperature | 10 | 350.4890 | 4.5603 |
| m26 | size at onset * water * temperature | 19 | 353.2056 | 7.2769 |
| m27 | size at onset * substrate * water * temperature | 52 | 359.3209 | 13.3922 |
| m3 | water | 4 | 362.7866 | 16.8579 |
| m7 | water + temperature | 5 | 362.9432 | 17.0145 |
| m11 | water * temperature | 7 | 366.1713 | 20.2426 |
| m5 | substrate + water | 7 | 367.7013 | 21.7726 |
| m8 | substrate + water + temperature | 8 | 367.9034 | 21.9747 |
| m24 | size at onset * substrate * water | 34 | 371.0130 | 25.0843 |
| m9 | substrate * water | 13 | 373.7902 | 27.8615 |
| m13 | size at onset | 4 | 377.7940 | 31.8653 |
| m16 | size at onset + temperature | 5 | 378.3645 | 32.4358 |
| m14 | size at onset + substrate | 7 | 381.4156 | 35.4869 |
| m23 | size at onset * temperature | 7 | 381.4607 | 35.5320 |
| m18 | size at onset + substrate + temperature | 8 | 381.8617 | 35.9330 |
| m12 | substrate * water * temperature | 25 | 383.8708 | 37.9421 |
| m21 | size at onset * substrate | 13 | 390.9331 | 45.0044 |
| m1 | NULL | 2 | 398.2626 | 52.3339 |
| m25 | size at onset * substrate * temperature | 23 | 398.4364 | 52.5077 |
| m4 | temperature | 3 | 398.7033 | 52.7746 |
| m2 | substrate | 5 | 403.5299 | 57.6012 |
| m6 | substrate + temperature | 6 | 403.9976 | 58.0689 |
| m10 | substrate * temperature | 9 | 407.6481 | 61.7194 |

**Supplementary Table 4** Leaf length model test table for the model m22; Signif. Codes: 0 '***', 0.001 '**', 0.01 '*', 0.05 '.', 0.1 ''; Residual standard error: 1.441 on 85 degrees of freedom; Multiple R-squared: 0.5166, Adjusted R-squared: 0.4711; F-statistic: 11.36 on 8 and 85 degrees of freedom, p-value <8.26E-11

| **variable** | **estimate** | **std. error** | **t-value** | **p-value** | **sign.** |
| --- | --- | --- | --- | --- | --- |
| (Intercept) | 2.8000 | 0.6442 | 4.346 | 3.82E-05 | *** |
| medium size of plant at onset | 0.4105 | 0.7241 | 0.567 | 0.57223 |  |
| medium size_of_plant at onset and floating leaves | 0.4000 | 0.9111 | 0.439 | 0.66176 |  |
| water level 1 cm | 0.7500 | 0.7890 | 0.951 | 0.34454 |  |
| water level 7 cm | 0.3667 | 1.0521 | 0.349 | 0.72831 |  |
| medium size * water level 1 cm | 0.5395 | 0.9241 | 0.584 | 0.56089 |  |
| medium size & floating leaves * water level 1 cm | 1.7500 | 1.2053 | 1.452 | 0.1502 |  |
| medium size * water level 7 cm | 1.8916 | 1.1601 | 1.631 | 0.10668 |  |
| medium size & floating leaves * water level 7 cm | 3.5762 | 1.2923 | 2.767 | 0.00694 | ** |
