# Supplementary figures and images for: Population genetics and plant growth experiments as prerequisite for conservation measures of the rare European aquatic plant Luronium natans (Alismataceae)
Source: Front Plant Sci. 2023 Jan 13;13:1069842. doi: 10.3389/fpls.2022.1069842 (PMC9880460; doi:10.3389/fpls.2022.1069842)

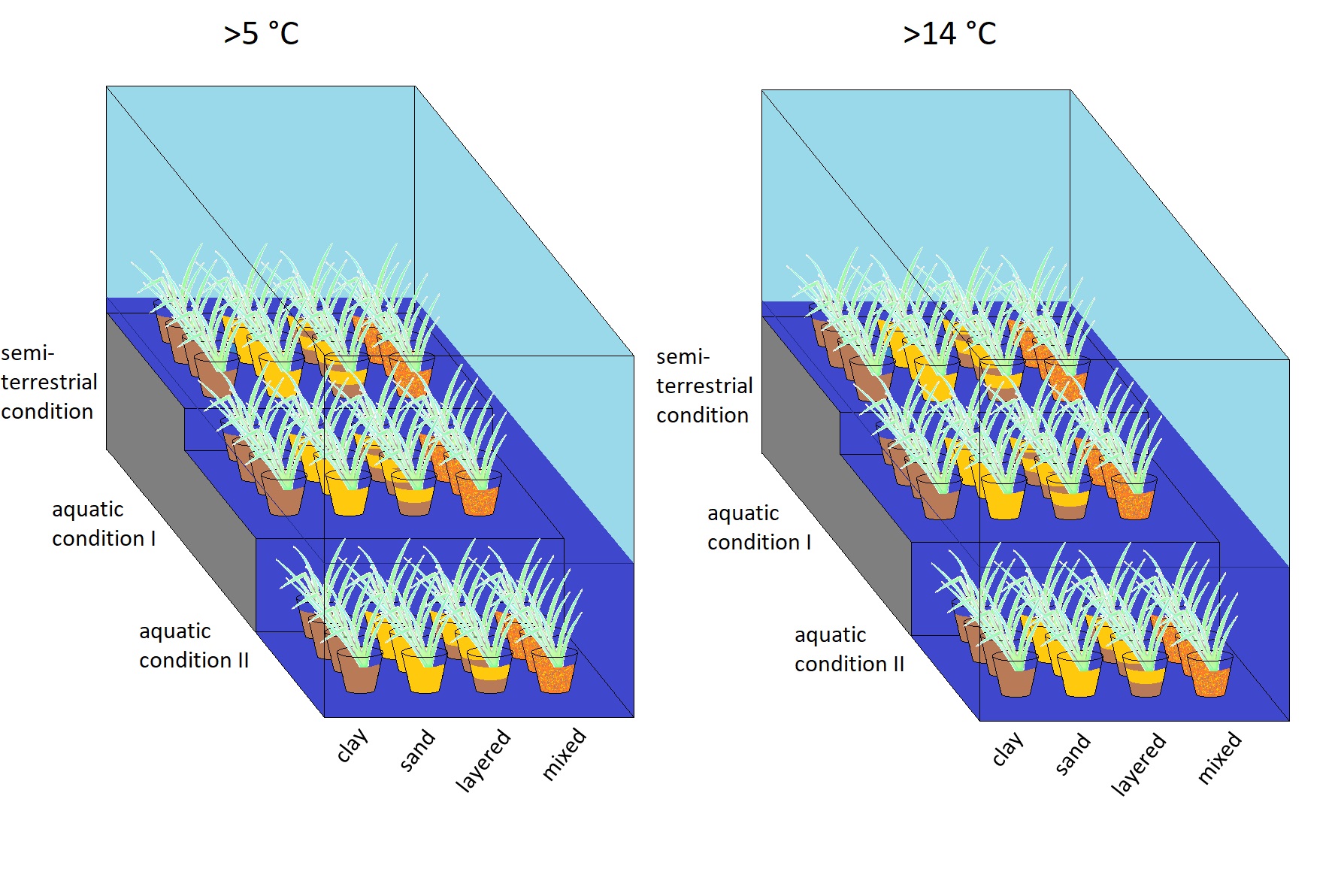

Supplement: Supplementary file 2 [file Image_1.jpeg]
